# Water-Airborne-Particle Abrasion as a Pre-Treatment to Improve Bioadhesion and Bond Strength of Glass–Ceramic Restorations: From In Vitro Study to 15-Year Survival Rate

**DOI:** 10.3390/ma14174966

**Published:** 2021-08-31

**Authors:** Luan Mavriqi, Francesco Valente, Bruna Sinjari, Oriana Trubiani, Sergio Caputi, Tonino Traini

**Affiliations:** 1 Department of Dentistry, Albanian University, 1001 Tirana, Albania; luanmavriqi@yahoo.com; 2 Department of Innovative Technologies in Medicine & Dentistry, University “G. d’Annunzio” of Chieti-Pescara, 66100 Chieti, Italy; francesco.valente@unich.it (F.V.); b.sinjari@unich.it (B.S.); oriana.trubiani@unich.it (O.T.); sergio.caputi@unich.it (S.C.); 3 Electron Microscopy Laboratory, University “G. d’Annunzio” of Chieti-Pescara, 66100 Chieti, Italy

**Keywords:** dental bonding, acid etching, air abrasion, microtensile bond strength, glass–ceramic restorations

## Abstract

The purposes of this study were to evaluate the efficacy of water–airborne-particle abrasion (WAPA) as pre-etching procedure for tooth surfaces to increase bond strength, and to compare the survival rate of WAPA vs. non-WAPA glass–ceramic restorations with a 15-year follow-up. The occlusal surfaces of 20 human molars were sectioned and flattened. The prepared surfaces areas were subdivided into two parts: one received WAPA treatment (prophy jet handpiece with 50 µm aluminium oxide particles) followed by acid etching (37% phosphoric acid for 20 s/3-step etch-and-rinse); the other one was only acid-etched. In total, 108 specimens were obtained from the teeth, of which 80 were used to measure the micro-tensile bond strength (μTBS) in the WAPA (n = 40) and control (n = 40) groups, while the remaining specimens (n = 28) were investigated via SEM to evaluate the micromorphology and roughness (*Ra*) before and after the different treatment steps. The survival rate (SR) was performed on 465 glass–ceramic restorations (131 patients) comparing WAPA treatment (n = 183) versus non-WAPA treatment (n = 282). The bond strength was 63.9 ± 7.7 MPa for the WAPA group and 51.7 ± 10.8 MPa for the control group (*p* < 0.001). The *Ra* was 98 ± 24 µm for the enamel control group, 150 ± 35 µm for the enamel WAPA group, 102 ± 27 µm for the dentin control group and 160 ± 25 µm for the dentin WAPA group. The *Ra* increase from the WAPA procedure for enamel and dentin was statistically significant (*p* < 0.05). Under SEM, resin tags were present in both groups although in the WAPA they appeared to be extended in a 3D arrangement. The SR of the WAPA group (11.4 years) was 94%, while the SR of the non-WAPA group (12.3 years) was 87.6% (*p* < 0.05). The WAPA treatment using aluminium oxide particles followed by a 3-step etch-and-rinse adhesive system significantly improved bioadhesion with an increased bond strength of 23.6% and provided superior long-term clinical performance of glass–ceramic restorations.

## 1. Introduction

Dental adhesive technology has had a great impact in direct and indirect restorative procedures, opening the way to metal-free adhesion and minimally invasive dentistry [1].

For direct restorations, all procedures are usually performed during the same appointment, whereas for indirect restorations a provisional phase is necessary. Delayed dentin sealing is traditionally performed for indirect restorations, so the dentin is sealed after the provisional phase during the cementation appointment. Unfortunately, this technique cannot provide optimal conditions for bonding procedures [2,3] due to tooth surface contamination by provisional cement, bacteria and even impression material [4]. To overcome these inconveniences and to improve the bonding performance, immediate dentin sealing (IDS) was introduced for adhesive restorations [4,5,6,7,8,9,10]. The IDS, consists of an adhesive procedure performed immediately after tooth preparation. This step has demonstrated improved adhesion [7].

Despite the good level of adhesion achieved for direct restorations and indirect ones with IDS, further improvements to dentin bonding are desirable because the final strength of the tooth-restoration complex is highly dependent on adhesive procedures [6].

For a physical phenomenon, a rougher dental surface may increase the adhesion of a restoration because it creates a more extended tooth–adhesive interface [11]. An intuitive method to achieve this could be tooth surface sandblasting [12,13]. Intraoral sandblasting with alumina particles (Al_2_O_3_) was first described in 1945 by Black [14]. Initially, it was reported that the bond strength to the tooth surface improved, also confirmed by recent investigations, and some authors adopted its use in clinical procedures even after preparing the cavity with rotating instruments [13,15,16,17,18]. Tooth sandblasting was therefore introduced in restorative dentistry as a method of cavity preparation and called “air abrasion” [19].

**Figure 1:**
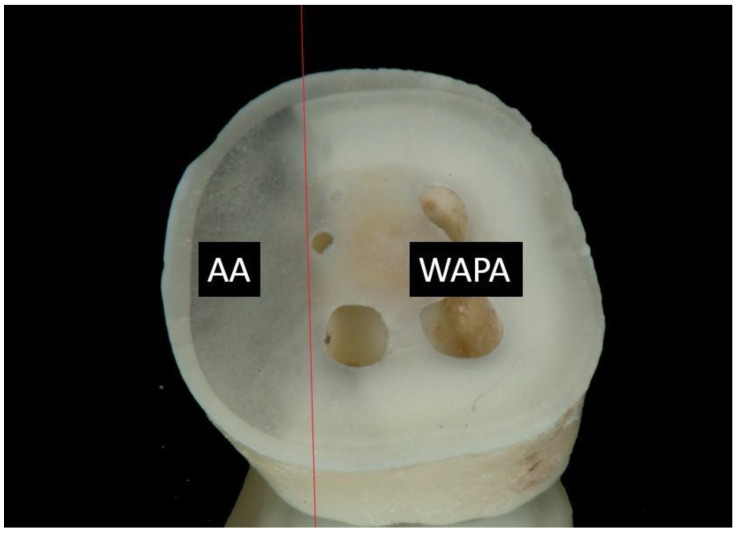
Image of a tooth surface treated with air abrasion (**AA**) and water–air-particle abrasion (**WAPA**). The red line delineates the boundary between the two treatments. AA shows the effects of black pigmentation of the dentin, while WAPA does not.

Despite these observations, the application of air abrasion in aesthetic restorative dentistry is still limited, probably related to the discolouring effects on the dentin. Unpublished observations showed that discoloration disappears if the tooth surface is treated with airborne-particle abrasion under a water jet (water–airborne-particle abrasion: WAPA) (Figure 1).

**Figure 2:**
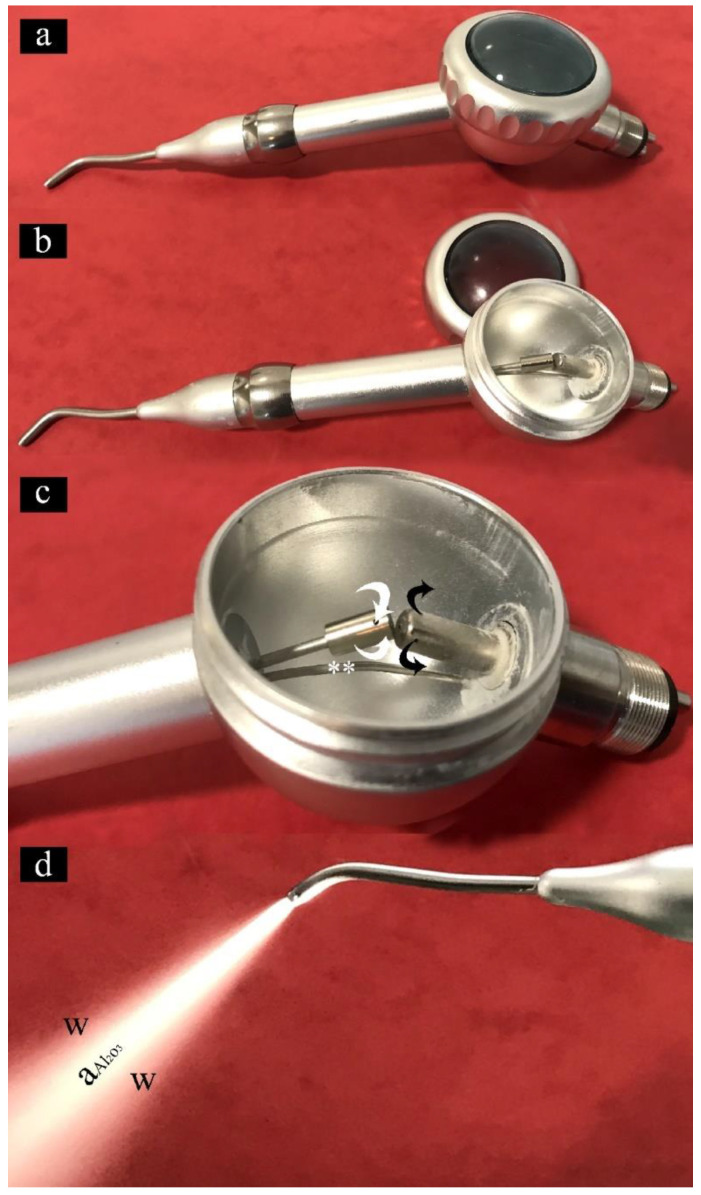
Images showing the main features of the prophy jet handpiece for the WAPA procedure: (**a**) external view of the prophy jet handpiece; (**b**) Al_2_O_3_ particle chamber opened; (**c**) functioning of the Al_2_O_3_ particle chamber. The black arrows show the flux of the incoming air; white arrows indicate the outgoing air enriched with Al_2_O_3_ particles. Water is carried with a tube (**) directly to the tip of the spout; (**d**) prophy jet handpiece in operation. From the spout of the handpiece an air-jet enriched with Al_2_O_3_ (aAl_2_O_3_) reaching approximately 400 km/h is wrapped in a stream of water (W).

WAPA is a clinical procedure carried out by means of a prophy jet handpiece (mounted on dental chair) applying water and powder directly onto the tooth surface. The tooth structure is conditioned using a stream of Al_2_O_3_ particles generated from compressed air and with aerosolized water. The prophy jet handpiece separates the air and water channels allowing the highly precise regulation of water and powder flow. By doing so, it produces effective kinetic energy for predictable treatment outcomes: water and powder meet upon impact with the tooth giving maximum efficiency and minimal aerosol dispersion. The abrasive particles strike the tooth with high velocity removing small amounts of tooth structure. The efficiency of removal is related to tissue hardness and the operating parameters of the device. Like air abrasion, several parameters such as air pressure (fixed on the chair standard at a value of 0.25 MPa), particle size (fixed at 50 µm), quantity of particles passing through the spout, handpiece spout diameter and angle, distance from the tooth (1.5–2 cm), and time of exposure (10–30 s) vary the quantity of tooth removal and depth of penetration [19]. Figure 2 shows the structure and the functioning of the prophy jet hand piece used to carry out the WAPA procedure.

Based on the above, this study was conceived to evaluate if WAPA pre-etching can provide superior bond strength and extend the clinical service of bonded ceramic restoration, without tooth blackening, with the purpose of associating it with the operative IDS protocol for biomimetic prosthetic restorations.

Therefore, this study compared the bond strength of an adhesive resin with WAPA followed by the 3-step etch-and-rinse procedure on the tooth surfaces (test) versus the conventional 3-step etch-and-rinse technique without WAPA (control). A null hypothesis (H_0_) of any difference in bond strength between the test and control groups was considered. Moreover, a retrospective clinical long-term evaluation was performed on 465 glass–ceramic restorations: 183 were placed using the improved IDS protocol (WAPA) and 282 using conventional IDS (non-WAPA), to verify if establishing WAPA procedure in clinical protocols leads to the better long-term success of bonded restorations.

## 2. Materials and Methods

**Figure 3:**
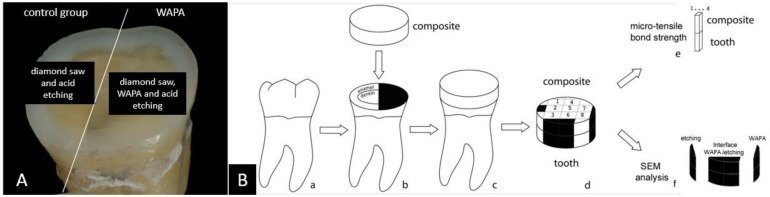
Specimen preparation and schematic drawing of the procedures. In (**A**) the flattened occlusal area of a specimen shows the distinction between the treatments. In (**B**), schematic drawings of each step a–f. From each tooth sample, only the best four specimens out of eight were selected (control group n = 2; WAPA group n = 2) for the microtensile bond strength test and three specimens for each tooth resulting from the cuttings (in black on d and f), were used for the SEM analysis.

Ethical approval was obtained from the Ethics Committee of the Albanian University, Albania (protocol code 278/3). Twenty unerupted human mandibular wisdom teeth (extracted for orthodontic reasons) were prepared following the technique developed by Shono et al. as shown in Figure 3 (Details can be found in [20]). The teeth were stored at 4 °C in a 0.15 M NaCl solution saturated with thymol and used within 1 month of extraction. Briefly, the occlusal surface of each tooth was cut and flattened to expose both the dentin and enamel, by means of a semi-automatic diamond saw cooled with running water (TMA2, Grottammare, Italy). The prepared teeth were cleansed in an ultrasonic bath of physiologic solution for 10 min at 40 °C. One-half of each flattened tooth surface was protected in a randomly chosen direction with a strip during the WAPA procedure. The teeth surfaces were then divided into two sectors, each one receiving a different treatment so that every tooth was used as its own control to overcome any statistical problem with power and number of specimens. From each tooth sample, eight specimens were obtained but only the best four were selected (control group n = 2; WAPA group n = 2) for the microtensile bond strength test. At the same time, three specimens for each tooth resulting from the cuttings were used for the SEM analysis (Figure 3B(d–f)).

**Figure 4:**
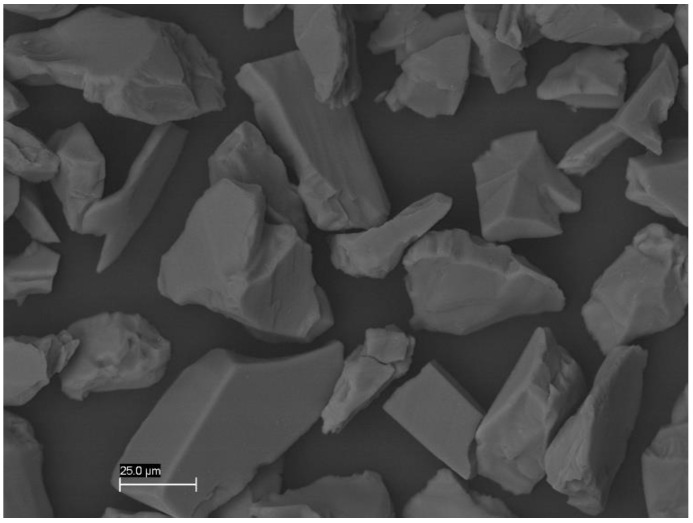
SEM image of Al₂O₃ particles of 50 µm that appeared in mean and with very irregular shapes. Mag. 1000×.

The WAPA procedure was carried out chairside by means of a prophy unit tooth polisher handpiece (Air Prophy Unit, Renfert GmbH, Hilzingen, Germany) with 50 μm aluminium oxide (Al₂O₃) particles at 0.25 MPa of air–water pressure, perpendicular to the surface (Figure 4). A working distance of 1.5 cm and time of action of 10 s were both standardized and controlled.

After treatment, the specimens were carefully cleansed and prepared for total etching and bonding. The 3-step etch-and-rinse adhesive system (OptiBond FL; Kerr, Orange, CA, USA) was used as follows: 20 s of etching with 37% phosphoric acid, abundant rinsing with water spray for 20 s, air drying for 5 s, application of primer (bottle 1) by means of microbrush (Micro Tip Applicator, GC Corp., Tokyo, Japan) with a light brushing motion for 10 s, air drying for 3 s, application of adhesive resin (bottle 2) with a light brushing motion for 10 s and air thinning for 3 s. The bonding resin was light cured with a multi-wave-length light-emitting diode curing lamp (Valo, Ultradent Products, South Jordan, UT, USA) for 40 s at 800 mW/cm² and with a wavelength of 395–480 nm at 3 mm tip-to-specimen distance. Finally, the crowns of the teeth were restored using a composite material (Z100; 3M ESPE, Seefeld, Germany) and then cut as previously described.

### 2.1. Microtensile Bond Strength (µTBS)

A total of 80 bar-shaped specimens with a bonding area of about 1 mm² were obtained (40 test and 40 for control) and used to test microtensile bond strength (µTBS) (Figure 3B(a–f)).

Before testing, each specimen was carefully measured with a digital calliper (Shimana SHAYDC082/83/84, Toronto, ON, Canada) to the nearest 0.01 mm for the cross-sectional area to calculate the results in MPa. The load at failure was recorded and the μTBS was measured through the testing machine (MTS810, MTS Co., Eden Prairie, MN, USA) at 0.5 mm/min crosshead speed. Specimens were fixed with adhesive cyanoacrylate (Super Attack; HENKEL Ag&Co KGaA, Düsseldorf, Germany) to the grips of the micro-tensile device.

### 2.2. Scanning Electron Microscopy (SEM)

After cleansing in an ultrasonic bath of distilled water, the specimens underwent critical point drying in Emitech K 850 (Emitech Ltd., Ashford, Kent, UK) and were then mounted onto aluminium stubs, sputter gold coated in Emitech K 550 (Emitech Ltd., Ashford, Kent, UK) and analysed via a scanning electron microscope (SEM) (Zeiss EVO 50 XVP, Carl Zeiss SMY Ltd., Cambridge, UK) equipped with a LaB₆ electron gun and an Everhart–Thornley tetra solid-state detector (4Q-BSD). SEM operating conditions included 5.0 kV accelerating voltage, 8.5 mm working distance and a 100 pA probe current for observations under variable pressure (0.75 torr). The images were captured with a line average technique using 20 scans.

#### SEM Analysis and Surface Roughness (Ra)

The microstructure morphology of the tooth surface at each step in both types of treatment was evaluated using additional specimens (n = 4). To evaluate the interface between bonding agent and hard tooth tissues qualitatively, specimens (n = 24) from the cuttings were used (Figure 3B(f)). SEM images of WAPA-treated vs. non-WAPA-treated enamel and dentin before and after etching as well as the micromorphology organization of the tooth–resin interface were obtained. Surface roughness (*Ra*) was measured on SEM images using Equation (1) for two-dimensional computation,

(1) $$Ra\left(z\right)=\frac{1}{n}\overset{n}{\underset{i=1}{\sum }}\left(z_{i}-\bar{z}\right)\;$$

as the roughness of the mean distance between the roughness profile and its mean line was reported in µm.

SEM stereo-imaging was used to reconstruct the surface topography. To obtain accurate results it was assured that brightness constancy and alignment were set in such a way that both images had approximately the same brightness and contrast.

In brief, stereo pair images were acquired with symmetrical tilt angles of −5° and +5°, and the elevation (relative to the centre of inclination on the specimen) was calculated as a function of the disparity. A horizontal disparity map was finally converted into heights according to the acquisition parameters––tilt angle, magnification, and pixel size––with simple trigonometric equations. Disparity *d* and Height *h* are related by Equation (2).

(2) $$d=2\;h\sin\left(\frac{\theta }{2}\right)$$

Therefore, the height *h* (in µm) of a point with a disparity *d* (in pixels) is related by Equation (3),

(3) $$h=\frac{dp}{2\sin\left(\frac{\theta }{2}\right)}$$

where *θ* is the total tilt angle, and *p* is the pixel size or the scale provided by the SEM system in µm.

### 2.3. Long-Term Survival Rate

The survival rate (SR) was performed on 465 glass–ceramic restorations on 131 patients (72 female, 59 male) between 2003 and 2018. The retrospective evaluation considered up to 15 years of follow-up. Causes of failure involved fracture and debonding or secondary caries, while abrasions were not considered among the complications.

There were no inclusion/exclusion criteria based on conditions related to the patients. Restorations were not classified for site of placement (anterior or posterior) material (feldspathic ceramic, lithium disilicate, zirconia-reinforced lithium silicate), restoration type (tooth crowns, veneers, inlays, onlays, overlays), or finishing line (e.g., rounded shoulder, chamfer). They were grouped only for different procedures: WAPA (n = 183) versus non-WAPA (n = 282). All the clinical and laboratory steps were performed by the same experienced operator (T.T.). The cementation protocol was performed with the materials and methods described in the previous in vitro part of this study, and under local anaesthesia and rubber dam isolation when necessary. After the teeth were prepared, in the WAPA group, IDS was performed with WAPA, acid etching and the 3-step etch-and-rinse adhesive procedure; in the control group, only acid etching and the 3-step etch-and-rinse adhesive procedure were performed. The oxygen inhibition layer (OIL) was removed with a mounted brush and prophy paste (Detrartrine, Septodont, Mataro, Spain) at low speed. The temporaries (crowns and veneers) were made of acrylic resin (ColdPac, Yates Motloid, Elmhurst, IL, USA) cemented with Temp Bond/Temp Bond Clear (Kerr, Orange, CA, USA) or made of temporary material (Fermit or Telio, Ivoclar Vivadent, Schaan, Liechtenstein) (inlays, onlays, overlays). The workflow was analogical for all the restorations.

Prior to cementation, the restorations were treated as follows:

- feldspathic ceramic: the bonding surface was etched with hydrofluoridic acid gel 9% (Porcelain Etch, Ultradent, South Jordan, UT, USA) for 1.30 min;
- lithium disilicate and zirconia-reinforced lithium silicate: the bonding surface was etched with hydrofluoric acid gel 4.5% (IPS Ceramic Etching Gel, Ivoclar Vivadent, Schaan, Liechtenstein) for 20 s.

The restorations were washed with distilled water, then the smear resulting from the acid etching was removed with an ultrasonic bath (Puresonic, Kiaccessori, Nola, Italy) in ethyl acetate for 5–10 min and the restoration was stored in ethyl alcohol 96–100% until silane application. Then a silane (Monobond S and Monobond Plus, Ivoclar Vivadent, Schaan, Liechtenstein) was applied by means of a microbrush for 60 s of action on the bonding surface of the restoration and let dry at room temperature. Right before cementation, the temporaries were removed, and the residual eliminated with a mounted brush and prophy paste at low speed. While the acid etching and adhesive procedures were re-performed on the tooth, the only bonding was applied with a microbrush on the bonding area of the restoration. The bonding on the restoration and tooth were not light cured: a thin layer of composite resin and a drop of flowable resin (to facilitate the flow) was applied to the restoration, and then the restoration was placed. To make the composite resin more fluid for proper cementation, the operator waited 1 min after placement to allow the body temperature to heat the composite to ca. 37 °C. Excess resin was continuously removed with a probe after the complete placement of the restoration. Then the composite was light cured from 3 different sides (palatal/lingual, buccal, occlusal), for 2 min per side. In the case of veneers, the first light-cured side was palatal/lingual; in the other cases, occlusal. Then the excess polymerized composite resin was gently removed with a curved lancet.

### 2.4. Statistical Analysis

Descriptive statistics were obtained for μTBS test data and for surface roughness data. The results were statistically inferred using the unpaired Student’s *t*-test for μTBS test data and a one-way ANOVA and Holm–Sidak method for multiple comparisons for surface roughness data. The Kaplan–Meier survival analysis was used to compare the mean survival rates between the two groups to determine whether the experimental treatment (WAPA) was an improvement over the traditional one (non-WAPA). Statistical significance was assayed using a Log Rank test after means calculation. The level of statistical significance was set at *p* < 0.05. The statistical analyses were performed using IBM SPSS Statistics v. 3.5 (IBM Corp, Armonk, NY, USA).

## 3. Results

Results of the microtensile bond strength (μTBS) test and roughness (*Ra*) analysis are collected in Table 1 and described in the following sections.

### 3.1. Microtensile Bond Strenght (µTBS)

**Figure 5:**
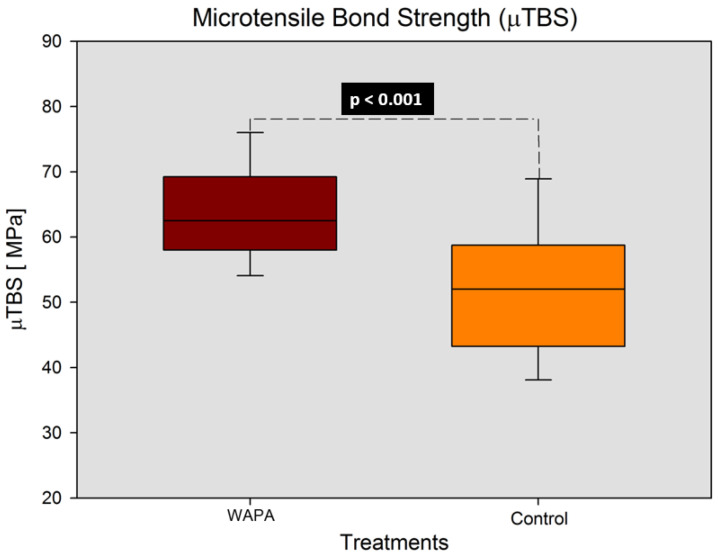
Results of the μTBS of the two different treatments (WAPA and control groups). WAPA: Water–airborne-particle abrasion. Statistically significant difference (*p* < 0.001) with unpaired *t*-test (power = 0.999 with α = 0.05).

The μTBS was 63.9 ± 7.8 MPa for the WAPA group and 51.7 ± 10.8 MPa for the control group. The difference was highly statistically significant (*p* < 0.001; power of the performed test = 0.999 with α = 0.05). Results are shown in Figure 5.

### 3.2. SEM Observations and Roughness Analysis (Ra)

**Figure 6:**
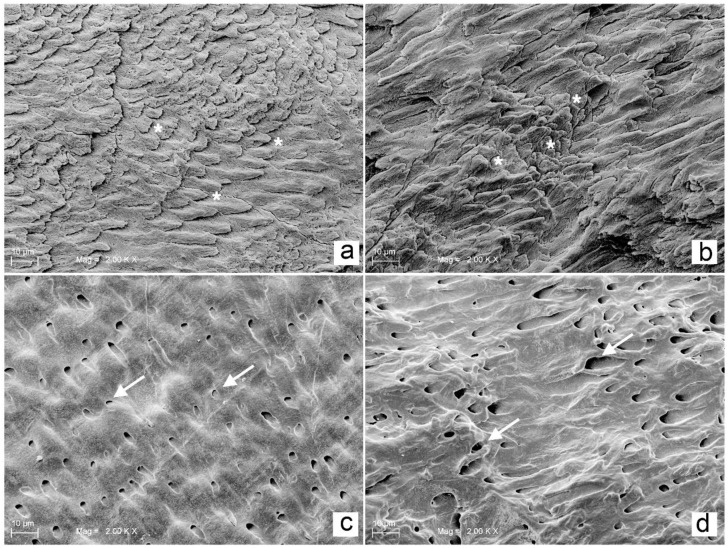
Comparison of enamel and dentin treated with acid-etching and WAPA and acid-etching procedures: (**a**) enamel acid-etched for 20 s, (*) rods of enamel prisms appear on the same level. Mag. 2000×; (**b**) enamel after WAPA and acid-etch treatment for 20 s, (*) rods of enamel prisms appear on different levels with a three-dimensional appearance. Mag. 2000×; (**c**) dentin after 20 s of etching, white arrows indicate open tubules which are located on the same level. Mag. 2000×; (**d**) dentin treated with WAPA followed by 20 s of etching, white arrows indicate open tubules which are located on different levels with a three-dimensional appearance. Mag. 2000×. After comparing the SEM images (**a**) vs. (**b**) and (**c**) vs. (**d**) it was possible to note that the main difference between specimens that were acid etched and those that underwent acid etching and WAPA was related to the 3D architecture, which was much more present in the WAPA specimens, both for enamel and dentin.

**Figure 7:**
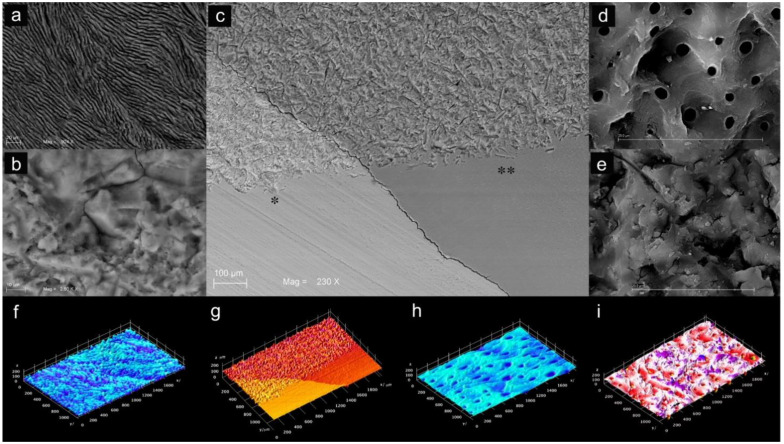
Comparison between SEM images of enamel and dentin after WAPA and acid-etching: (**a**) enamel prisms after WAPA procedure and acid-etching. Mag. 924×; (**b**) enamel after WAPA procedure before acid-etching. The WAPA procedure introduce a 3D dimension to the surface. Mag. 2500×; (**c**) enamel (*) and dentin (**) with WAPA procedure (top side) and without WAPA procedure (bottom side). Mag. 230×; (**d**) dentin after WAPA procedure and acid-etching. Several dentin tubules appeared open on dentin surface with different directions. Mag 2500×; (**e**) dentin after WAPA procedure before acid-etching. Mag. 4000×; (**f**) 3D reconstruction of enamel roughness after WAPA and acid etching procedures. The *Ra* was 150 ± 35 µm; (**g**) 3D reconstruction of the surface roughness of enamel (brighter orange side) and dentin (darker orange side) treated with WAPA procedure (top side) and without WAPA procedure (bottom side); enamel treated with only acid etching showed a *Ra* of 98 ± 24 µm; (**h**) 3D reconstruction of the surface roughness of dentin treated only with acid-etching. The *Ra* was of 102 ± 27 µm; (**i**) 3D reconstruction of the surface roughness of dentin after WAPA and acid etching procedures. The surface *Ra* was of 160 ± 25 µm.

**Figure 8:**
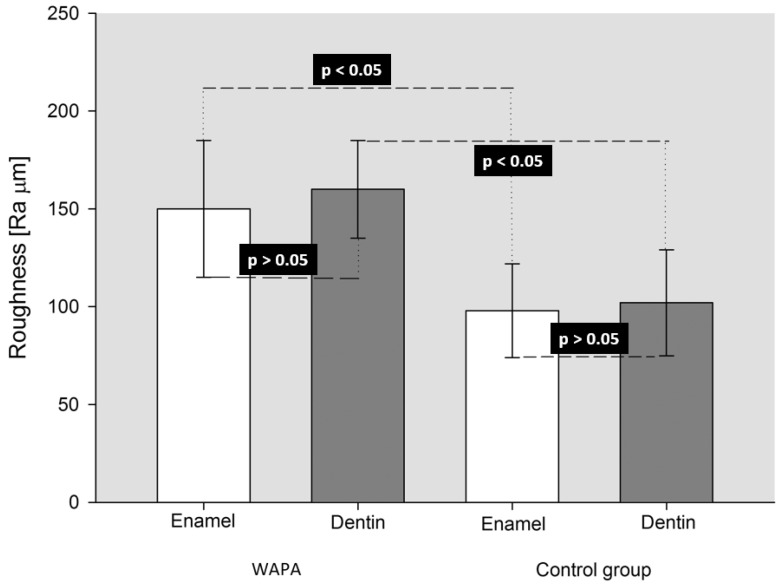
Results of One-way ANOVA (*p* = 0.004; power = 0.881 with α = 0.05) and Holm–Sidak pairwise multiple comparison procedures for *Ra* among both treatments and tissues. WAPA: water–airborne-particle abrasion and acid-etching; Control group: acid-etching only. A statistically significant difference (*p* < 0.05) was present between WAPA and Control group for both enamel and dentin tissues. No statistically significant difference (*p* > 0.05) was present between different tissue in the same group of treatment.

The effects of the WAPA procedure on tooth structures (enamel and dentin) before any adhesive procedure are shown in Figure 6 and Figure 7. The surfaces treated with WAPA followed by acid etching appeared to be rougher and more complex (Figure 6b,d) compared to the surfaces that were only acid etched (Figure 6a–c and Figure 7c). Enamel without treatment showed an *Ra* of 98 ± 24 µm, but after the WAPA treatment the *Ra* was 150 ± 35 µm (Figure 7f,g). Dentin without treatment showed a mean *Ra* of 102 ± 27 µm, while after WAPA treatment it was 160 ± 25 µm (Figure 7h,i). The increase in surface roughness due to WAPA on both enamel and dentin was statistically significant (*p* < 0.05), and the mean was higher by about 54%. At the same time, no statistically significant differences (*p* > 0.05) were detected for intragroup comparisons either for enamel and dentin before or after the treatments (Figure 8). After etching, both the enamel and dentin showed differences between the two groups for qualitative 3D-aspects of the surfaces due to the increased roughness. In fact, in the WAPA group, the enamel prisms were more markedly interrupted and the dentinal tubules appeared to be open in different planes of orientation with a “canyon” aspect when compared to the control group (Figure 6).

**Figure 9:**
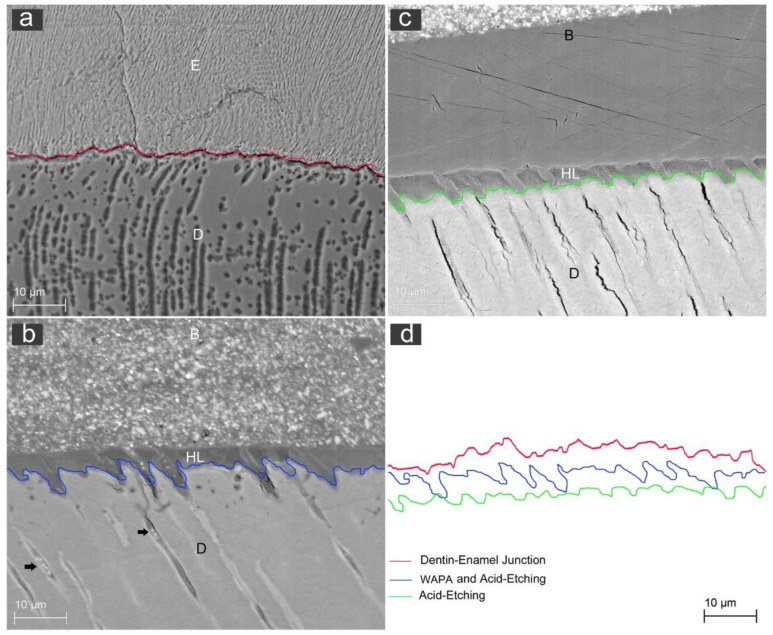
Representative SEM images for the qualitative evaluation of line profile at the interface between dentin and resin: (**a**) red-line profile of the dentin/enamel junction (DEJ) of a sound tooth; (E) enamel; (D) dentin. Mag 500×; (**b**) blue-line profile of the interface between dentin and resin for the WAPA group. (B) resin; (HL) hybrid layer; (D) dentin; black arrows: resin tags inside dentinal tubules. Mag 500×; (**c**) green-line profile of the interface between dentin and resin for the control group. (B) resin; (HL) hybrid layer; (D) dentin. Mag 500×; (**d**) comparison among line profiles. The similarity between the red and blue profile lines and the substantially different trend for the green line appears evident.

**Figure 10:**
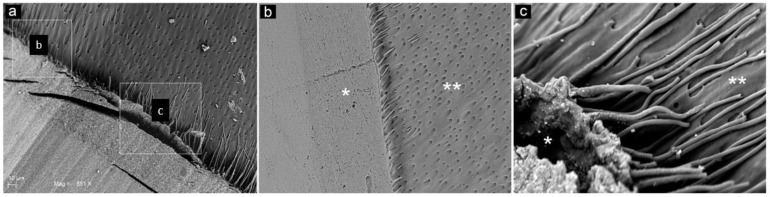
Representative SEM observations of the tooth-restoration interface for both WAPA and control groups: (**a**) SEM image of both types of preparation are visible: the WAPA is identified by square **c**, while control is identified by square (**b**). Mag. 851×; (**b**) Higher magnification of control group (3000×). At the interface, between dentin (**) and the restoration material (*) the resin tags appear to be shorter and less developed if compared with those of (**c**); (**c**) Higher magnification of the WAPA group (4000×). At the interface, between dentin (**) and restoration material (*), the resin tags appeared to be more extended inside the dentin tubules.

After the restorative procedures, the differences between the two groups were mainly visible in the dentin, where resin tags appeared (Figure 9 and Figure 10). The WAPA group interface profile appeared to be much more extended for size, length and a more complex spatial arrangement than the one in the control group (Figure 9b,c). The qualitative analysis of the surface profile at the dentin/bonding interface compared to the dentin/enamel junction (DEJ) demonstrated a similarity between the WAPA and DEJ (Figure 9d and Figure S1). However, in both WAPA and the control groups there was intimate contact between the restorative material and the tooth.

Analysing the resin tag disposition at the interface between the restoration material and dentin in both the WAPA and control group, it is possible to note that in the control group they were shorter and more irregularly arranged than in the WAPA group, where they protruded to greater extent into the dentinal tubules (Figure 10 and Figure S2).

### 3.3. Long-Term Survival Rate

**Figure 11:**
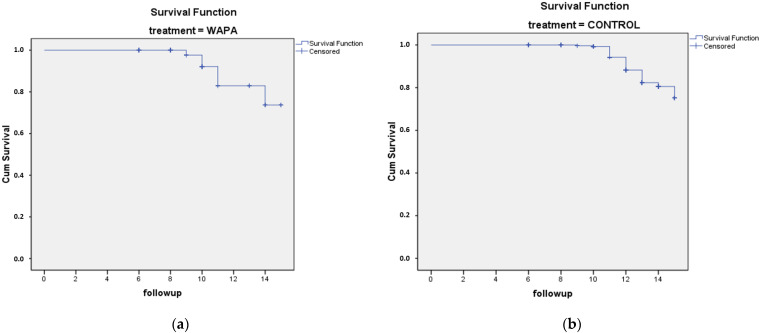
Kaplan–Meier survival analysis of 465 glass–ceramic restorations (131 patients, 59 males, 72 female) for up to 15 years (2003–2018): (**a**) WAPA treatment, n = 183; (**b**) control (non-WAPA treatment), n = 282. For both graphs, the *y* axis is cumulative survival (%), and the *x* axis is follow-up (years). The difference was statistically significant (Log Rank test, *p* < 0.05).

The SR for the WAPA group at 14.1 years was 94%, while the SR for the non-WAPA group at 14.4 years was 87.6%. Mean calculation is shown in Table 2. A difference of 6.4% was statistically significant (*p* < 0.05) (Figure 11).

## 4. Discussion

As stated previously, the novelty of the present study was to test and demonstrate after a lengthy time the clinical efficacy of the WAPA procedure when associated with the IDS protocol for bonded ceramic restorations. For this reason, this study consisted of two parts: the first, an in vitro study, and the second, an in vivo evaluation after a lengthy time. We aimed to integrate the laboratory and clinical results for a higher level of evidence to overcome the widespread dichotomous behaviour that separates in-vitro evidence from clinical applications.

The results of the in vitro part of the study rejected the hypothesis under test. The WAPA procedure led to an increase of the bond strength between the restoration and the tooth of 12.2 MPa (23.6%). This improvement was statistically significant (*p* < 0.001) and associated with an increase in the roughness at the tooth tissues/resin interface of about 54%, which was also statistically significant (*p* < 0.05).

Our results agreed with the majority of the studies that tested sandblasting on tooth surfaces [15,17,18,20,21]. However, some authors reported no difference in bond strength between sandblasted and non-sandblasted teeth although they used self-etching adhesives instead of etch-and-rinse adhesives, as we did, and detected lower μTBS values [22,23]. This methodological difference could be relevant since some authors reported that sandblasting increased the bond strength of a restoration only if it were associated with a total etching procedure [19,21]; however, resin tags without WAPA do not contribute to dentin adhesion in self-etching adhesives [24]. These topics are of extreme importance since the present SEM qualitative analysis showed that the enamel surface of the WAPA group was generally more irregular, while the dentin surface showed more open dentinal tubules (Figure 6). At the interface between the tooth and the restoration, the resin tags were more extended inside the dentin tubules, and this survey could be, in our opinion, the key factor to explaining the major bond strength of the tooth restoration (Figure 10). Thus, since 3-step adhesive systems perform the best and still are the gold standard for cementing indirect restorations [25], even more so when WAPA is performed, it is better to carry out a total etching adhesive procedure rather than a self-etching one to generate more open dentinal tubules and provide longer resin tags that can provide superior microtensile bond strength. It would be desirable if future investigations focused on the correlation among resin tag number, length, and disposition in influencing bond strength after WAPA.

Our observations on surface roughness are in agreement with Patcas et al., who reported a rougher enamel surface after sandblasting and acid etching compared to etching procedure alone [26]. Nevertheless, our results disagreed with those of Chinelatti et al., who reported no differences in enamel between sandblasting and sandblasting followed by acid etching. Regarding the effects of sandblasting on dentin, the same authors reported, according to our study, that dentinal tubules were more open when the acid etching procedure was carried out after sandblasting [17]. Although not statistically significant, it is interesting to note that, as expected, after WAPA the dentin *Ra* (160 ± 25 µm) was higher than the enamel *Ra* (150 ± 35 µm), principally because of its less tough structure. On the other hand, the enamel prisms and mature matrix offered superior wear resistance to high-velocity sandblasting with Al_2_O_3_ 50 µm particles.

The major limitation of the in vitro part of the present study is related to the absence of dentinal fluid pressure. However, this limitation was overcome by the results obtained from the in vivo part since it is a mere simulation of reality. It demonstrated that glass–ceramic restoration cemented with WAPA has a superior SR than restoration without WAPA for up to 15 years of service. The difference of 6.4% was statistically significant (*p* < 0.05). To the knowledge of the authors, this is the first study to evaluate the clinical performance of a considerable number of glass–ceramic restorations following a WAPA procedure with a long-term follow up; hence, the outcomes are highly valuable. The clinical results confirmed the results of the in vitro part of the study, validating the cementation protocol used in the study and nullifying potentially affecting variables. It must be noted that survival analysis was performed only on groups where WAPA was performed or not. It therefore would be an additional benefit for future evaluations to analyse groups with different selection criteria, such as patient-related conditions (e.g., bruxism), base material of the restoration (feldspathic ceramic, lithium disilicate, zirconia-reinforced lithium silicate) or kind of restoration (tooth crowns, veneers, inlays, onlays, overlays), to learn more about the impact of WAPA in specific clinical situations.

From a clinical point of view, it can be stated that the WAPA procedure is a useful tool in minimally invasive dentistry to prevent tooth blackening and improve the adhesion of the bonded restorations to sclerotic dentin, which is usually problematic. However, these speculations need further scientific investigations.

## 5. Conclusions

The results of this study indicated that tooth surfaces treated with 50 μm of aluminium dioxide WAPA and a 3 step etch-and-rinse adhesive procedure had increased surface roughness and an microtensile bond strength of 12.2 MPa (23.6%) without detrimental tooth blackening. These outcomes were positively confirmed in the clinical scenario, where glass–ceramic restorations cemented with an operative IDS protocol with WAPA showed superior long-term clinical performance than when WAPA was not performed. Therefore, WAPA can be considered an effective pre-etching procedure associated with the operative IDS protocol for the long-term success of biomimetic prosthetic restorations in clinical dentistry.

## Figures and Tables

**Table 1:** Descriptive statistics of microtensile bond strength (μTBS) and roughness (*Ra*) between WAPA group (water–airborne-particle abrasion and acid-etching) and control group (only acid-etching).

	μTBS ^a^		Roughness ^b^			
	**WAPA**	**Control**	**WAPA**		**Control**	
			**Dentin**	**Enamel**	**Dentin**	**Enamel**
Mean	63.9	51.7	160	150	102	98
SD	7.8	10.8	25	35	27	24

SD: standard deviation; ^a^ MPa; ^b^ *Ra* (µm).

**Table 2:** Means calculation for clinical WAPA and non-WAPA groups.

Treatment	Mean ^1^			
	Estimate	SE	CI 95%	
			LB	UB
WAPA	14.12	0.29	13.56	14.68
non-WAPA	14.44	0.09	14.27	14.61
Overall	14.34	0.09	14.17	14.52

^1^ Estimation is limited to the largest survival time if it is censored. SE: standard error; CI 95%: 95% confidence interval; LB: Lower Bound; UB: Upper Bound. Data are expressed in years.

## Data Availability

Data are available upon request to the corresponding author.

## Supplementary Materials

The following are available online at https://www.mdpi.com/article/10.3390/ma14174966/s1, Figure S1: SEM image showing the dentin-enamel junction architecture between dentin (D) and enamel (E) at high magnification. SEM, Mag. 10k×, Figure S2: Additional SEM image of the interface between dentin (D) and resin (B) of the WAPA group: it is possible to note the resin tags departing form the resin and the hybrid layer (HL) and developing into the dentinal tubules (black arrows). Mag. 4400×.
