# Supplementary material for: Water-Airborne-Particle Abrasion as a Pre-Treatment to Improve Bioadhesion and Bond Strength of Glass–Ceramic Restorations: From In Vitro Study to 15-Year Survival Rate
Source: Materials (Basel). 2021 Aug 31;14(17):4966. doi: 10.3390/ma14174966 (PMC8433990; doi:10.3390/ma14174966)
Supplement: Supplementary file 1 [file materials-14-04966-s001.zip › Supplementary Materials.final.pdf]

*materials*

MDPI

*Article*

# Water-Airborne-Particle Abrasion as a Pre-Treatment to Improve Bioadhesion and Bond Strength of Glass–Ceramic Restorations: From In Vitro Study to 15-Year Survival Rate

**Luan Mavriqi [1], Francesco Valente [2,3], Bruna Sinjari [2,3], Oriana Trubiani [2], Sergio Caputi [2,3] and Tonino Traini [2,3,*]**

[1] Department of Dentistry, Albanian University, 1001 Tirana, Albania; luanmavriqi@yahoo.com
[2] Department of Innovative Technologies in Medicine & Dentistry, University "G. d'Annunzio" of Chieti-Pescara, 66100 Chieti, Italy; francesco.valente@unich.it (F.V.); b.sinjari@unich.it (B.S.); oriana.trubiani@unich.it (O.T.); sergio.caputi@unich.it (S.C.)
[3] Electron Microscopy Laboratory, University "G. d'Annunzio" of Chieti-Pescara, 66100 Chieti, Italy
* Correspondence: tonino.traini@unich.it; Tel.: +39-08713554143

## Supplementary Materials

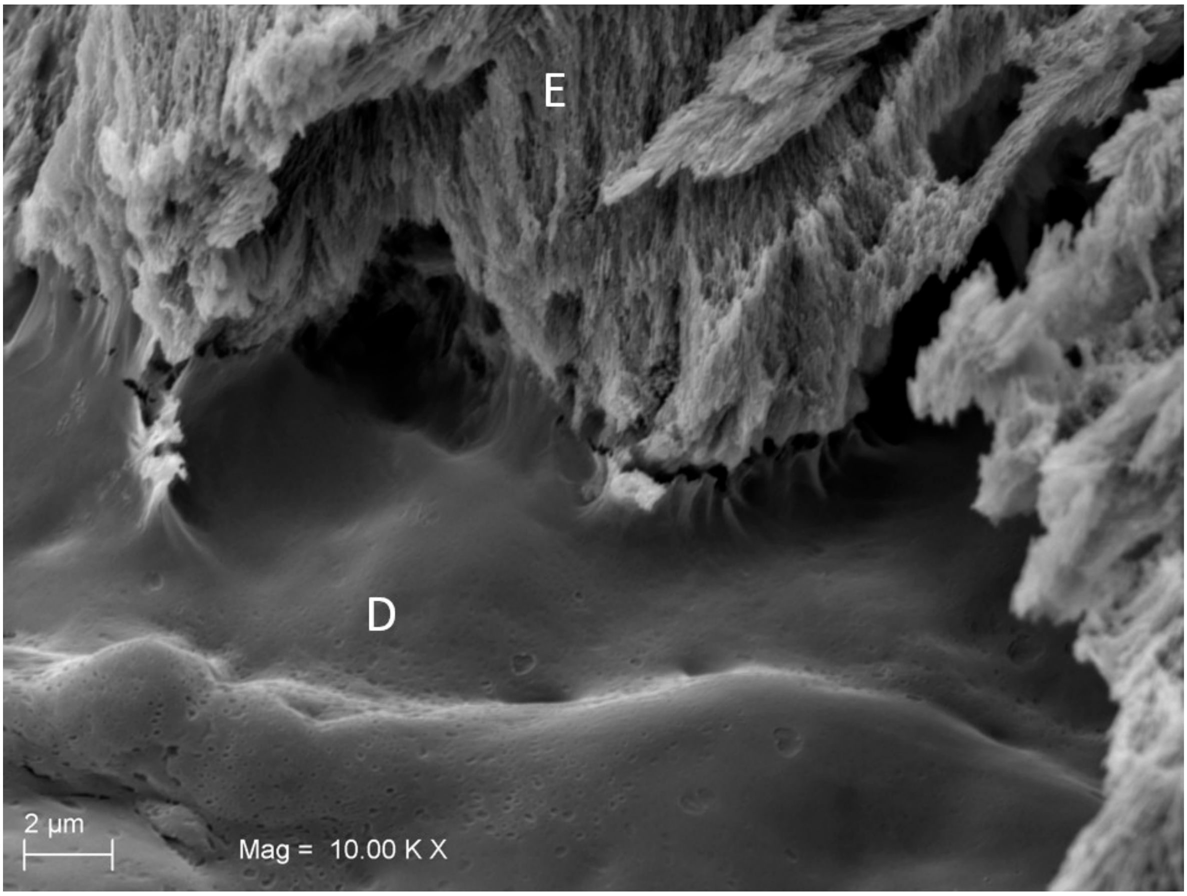


**Figure S1.** SEM image showing the dentin-enamel junction architecture between dentin (D) and enamel (E) at high magnification. SEM, Mag. 10kx.

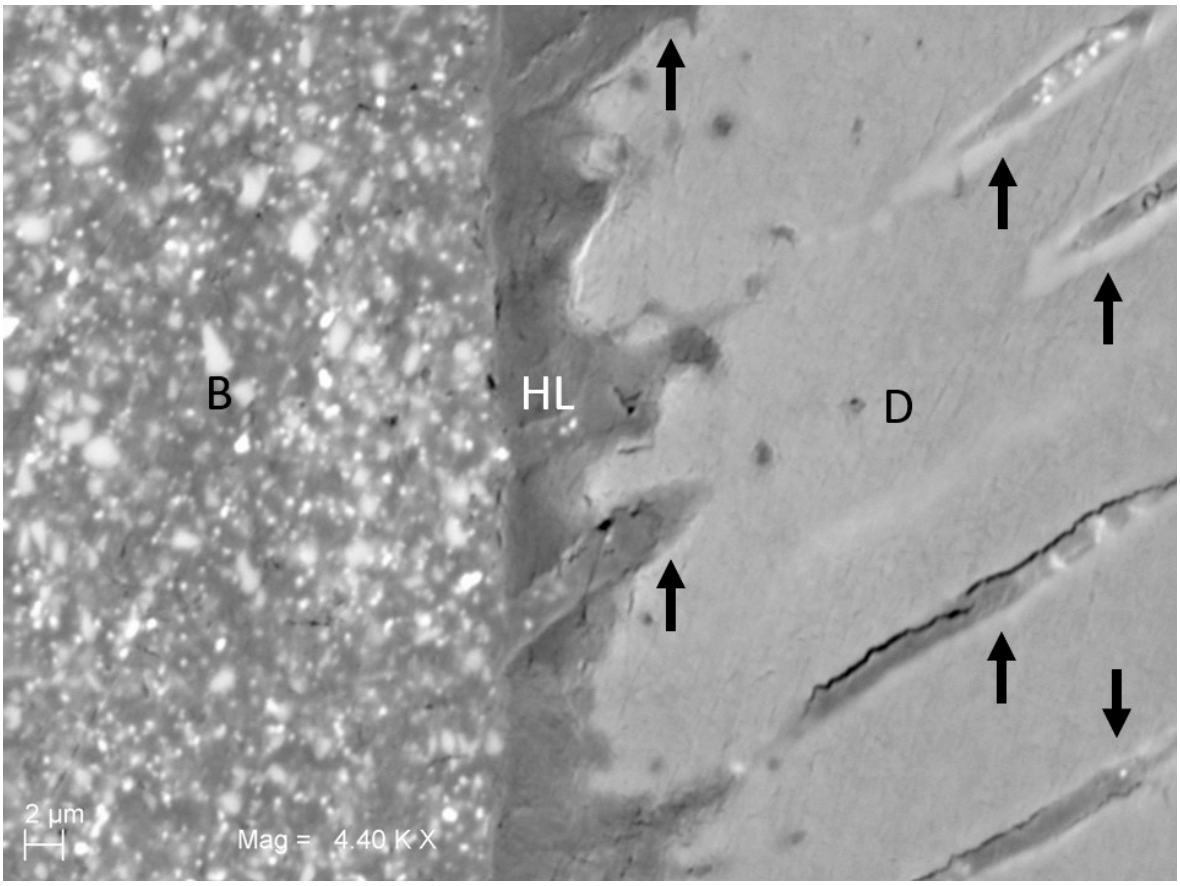


**Figure S2:** Additional SEM image of the interface between dentin (D) and resin (B) of the WAPA group: it is possible to note the resin tags departing form the resin and the hybrid layer (HL) and developing into the dentinal tubules (black arrows). Mag. 4400x.
